# Association between anthropometric markers of adiposity, adipokines and vitamin D levels

**DOI:** 10.1038/s41598-022-19409-9

**Published:** 2022-09-14

**Authors:** Pollyanna Patriota, Serge Rezzi, Idris Guessous, Pedro Marques-Vidal

**Affiliations:** 1 Swiss Nutrition and Health Foundation, 1066 Epalinges, Switzerland; 2 Division of Primary Care Medicine, Department of Primary Care Medicine, Geneva University Hospitals, Geneva, Switzerland; 3 Department of Medicine, Internal Medicine, Lausanne University Hospital, University of Lausanne, Office BH10-642, 46 Rue du Bugnon 46, 1011 Lausanne, Switzerland

**Keywords:** Health care, Nutrition

## Abstract

Inverse association between serum levels of vitamin D and obesity has been pointed out in several studies. Our aim was to identify to the associations between vitamin D levels and a large panel of anthropometric markers and adipokines. Cross-sectional study including 6485 participants. Anthropometric markers included body mass index (BMI), % body fat, waist, waist-to-hip (WHR), waist-to-height (WHtR), conicity index, body roundness index (BRI) and a body shape index (ABSI). 55.7% of women and 60.1% of men presented with vitamin D deficiency. Vitamin D levels were negatively associated with most anthropometric markers, with correlation coefficients ranging between −0.017 (ABSI) and −0.192 (BMI) in women and between −0.026 (weight) and −0.130 (% body fat) in men. Vitamin D levels were inversely associated with leptin levels in both sexes and positively associated with adiponectin levels in women only. The likelihood of vitamin D deficiency increased with increasing adiposity levels, except for ABSI (women) and BMI (men). Total body fat, rather than localized or unevenly distributed body fat, is the adiposity marker most associated with decreased vitamin D levels. Monitoring vitamin D levels in people with overweight/obesity is essential.

## Introduction

Vitamin D deficiency is common among adults^1^. The causes for vitamin D deficiency include reduced ability to synthesize vitamin D in the skin due to reduced sun exposure or skin pigmentation, decreased vitamin D dietary intake and or intestinal absorption, and increased adiposity^2–4^. The mechanisms associating increased adiposity (obesity) and vitamin D insufficiency are poorly understood; the main hypothesis is the sequestration of vitamin D by the adipose tissue, as suggested by the well reported negative association between serum vitamin D levels and fat mass in obesity^5^. In the Swiss Salt study, a one-unit increase in BMI was associated with an 8% decreased likelihood of being in the highest tertile of vitamin D^3^. Several systematic reviews and meta-analyses found inverse associations between vitamin D levels and waist circumference^6^ or fat mass^7^. However, most studies assessing the association between vitamin D and obesity focused on a single anthropometric marker. Several obesity markers such as waist circumference, body composition, the Valdez conicity index, the body roundness index (BRI) and a body shape index (ABSI)^8–10^ exist, but their joint analysis has seldom been conducted.

Some studies suggest that vitamin D modulates the secretions of many of these adipokines^11^.The Leptin has been identified as a marker that can be influenced by vitamin D levels, but the mechanisms that explain this association are still controversial^11,12^.

In this study, we aimed at assessing the associations between vitamin D levels and a large panel of anthropometric markers and adipokines, in a cross-sectional population-based study.

## Participants and methods

### Study design

The CoLaus (*Co*horte *Laus*annoise) study is a population-based prospective study assessing the clinical, biological, and genetic determinants of cardiovascular disease aged 35 to 75 years at baseline, living in the city of Lausanne, Switzerland)^13^. In each survey, participants answered questionnaires, underwent a clinical examination and blood samples were drawn for analyses^13^. Recruitment began in June 2003 and ended in May 2006. For this cross-sectional analysis, all participants at were eligible and no control group was created.

### Anthropometry

Anthropometric measurements were conducted using a standard methodology. Body weight and height were measured with participants barefoot and in light indoor clothes. Body weight was measured in kilograms to the nearest 100 g using a Seca® scale (Hamburg, Germany). Height was measured to the nearest 5 mm using a Seca® (Hamburg, Germany) height gauge^14^. Body mass index (BMI) was computed and categorized into underweight (< 18.5 kg/m^2^), normal (18.5–24.9 kg/m^2^), overweight (25–29.9 kg/m^2^) and obesity (≥ 30 kg/m^2^)^15^.

Waist circumference (WC) was measured mid-way between the lowest rib and the iliac crest, and hip was measured at the largest location, using a non-stretchable tape; the average of two measurements was taken^14^. Abdominal obesity was defined as a waist circumference > 102 cm (men) or > 88 cm (women). A high waist to height ratio (WHtR) was defined as > 0.5^16^; the WHtR is considered as a good indicator of abdominal obesity^17^.

Fat and fat-free mass (in percent of total body weight) were assessed by electrical bioimpedance in the lying position after a 5-min rest using the Bodystat® 1500 body mass analyzer (Bodystat Ltd, Isle of Man, England)^18^. This device has been shown to correlate well (r = 0.968) with measurements from dual energy X-ray absorptiometry (DEXA)^18^.

The conicity index (CI) was calculated according to Valdez^9^. It is based upon the idea that people accumulate fat around the waist and, the shape of their bodies seems to change from that of a cylinder to that of a “double cone” (two cones with a common base). The CI is determined by the formula

$$CI=\frac{Waist}{0.109\times \sqrt{\frac{Body\, weight}{Height}}}$$

The CI was further categorized as normal if < 1.25 and < 1.18 for men and women, respectively, and as high if ≥ 1.25 and ≥ 1.18 for men and women, respectively^19^.

Body roundness index (BRI)^20^ was computed according to and is based on waist circumference and height.

$$BRI=364.2-365.5\times \sqrt{1-\left[\frac{{\left(\frac{Waist}{2\pi }\right)}^{2}}{{\left(0.5\times Height\right)}^{2}}\right]}$$

A Body shape index (ABSI)^21^ was computed according to and is based on waist, BMI and height. Since there are no clinical cutoff values for BRI or ABSI, high BRI and ABSI were defined as those within the highest quartile group (Q4).

$$ABSI=\frac{Waist}{{BMI}^{2/3} \times {Height}^{1/2}}$$

### Vitamin D levels

Vitamin D was assessed at baseline through an ultra-HPLC tandem-MS system. The calibrators, 3Plus1 Multilevel Serum Calibrator Set 25-OH-Vitamin D3/D2 (ChromoSystems), were standardized against the National Institute of Standards and Technology 972 reference material. Serum 25(OH)D3 and 3-epi-25(OH)D3 were expressed in nanomoles per liter (conversion factor: 1 nmol/L = 0.4006 μg/L). The interday CV% was 4.6% at 40 nmol/L^22^. Vitamin D levels were further categorized as normal (≥ 30 ng/mL or ≥ 75 nmol/l), insufficiency (21 to 29 ng/mL or 50–75 nmol/l) and deficiency (< 20 ng/mL or < 50 nmol/l)^23^. Hypovitaminosis D was defined for vitamin D levels < 30 ng/mL or < 75 nmol/l, encompassing insufficiency plus deficiency^24^.

### Other covariates

Educational level was categorized into university, high school, apprenticeship, and mandatory. Nationality as born in Switzerland or not. Smoking status was self-reported and categorized as never, former, and current. Physical activity was considered if the participant reported performing at least twice a week a minimum of 20 min of leisure-time physical activity^13^.

Adipokines (adiponectin and leptin) were assessed at baseline. Adiponectin was assessed by ELISA (R&D Systems, Inc, Minneapolis, USA), with a maximum inter-assay CV of 8.3% and a maximum intra-assay CV of 8.3%. Leptin was assessed by ELISA (American Laboratory Products Company, Windham, USA) with a maximum inter-assay CV of 12.8% and a maximum intra-assay CV of 5.8%. High sensitive C-reactive protein (CRP) was assessed by immunoassay and latex HS on a Modular P apparatus (Roche Diagnostics, Basel, Switzerland).

### Exclusion criteria

Participants were excluded if they lacked any variable needed for the bivariate and the multivariate analyses. Hence, participants devoid of vitamin D data; without anthropometric measurements and any covariate needed for adjustment (education, smoking, BMI, or physical activity) were excluded. The exclusion procedure was conducted sequentially as follows: first, participants devoid of vitamin D data were excluded; of the remaining participants, those without anthropometric measurements were excluded; finally, of the remaining participants, those missing any covariate were excluded.

### Statistical analysis

Statistical analyses were performed using Stata version 16.1 for Windows (Stata Corp, College Station, Texas, USA)^25^. Descriptive results were expressed as number of participants (percentage) for categorical variables and as average ± standard deviation or median [interquartile range] for continuous variables.

As adiposity measures differ between sexes, stratification on the latter was performed. The associations between vitamin D levels and anthropometric markers (BMI, waist, WHR, WHtR, %fat as assessed by bioimpedance, CI, BRI and AABSI) were assessed as follows. First, comparison of vitamin D levels according to categories of anthropometric markers was performed using one-way (bivariate) or multivariate analysis of variance. Multivariate analysis was adjusted for age (continuous), nationality (Swiss, other), month, smoking categories (never, former, current), vitamin D supplementation (yes, no) and physical activity (yes, no) and the results were expressed as adjusted mean ± standard error. Second, bivariate nonparametric Spearman correlations and their 95% CIs were calculated between vitamin D levels and anthropometric markers as continuous variables. A stepwise multivariate linear regression analysis with age (continuous), nationality (Swiss, other), month, smoking categories (never, former, current), vitamin D supplementation (yes, no) and physical activity (yes, no) as locked terms was conducted to identify the anthropometric marker most associated with vitamin D levels. For simplicity, all anthropometric markers were standardized (i.e., zero average and unit standard deviation) before the stepwise regression.

Third, the association between vitamin D deficiency and categories of anthropometric markers was assessed using chi-square (bivariate analysis) and multivariate logistic regression with vitamin D deficiency (yes, no) as the dependent variable and adjusting for age (continuous), nationality (Swiss, other), month, smoking categories (never, former, current), vitamin D supplementation (yes, no) and physical activity (yes, no).

Sensitivity analyses were conducted by excluding participants receiving medically prescribed vitamin D levels. Finally, the analysis of the associations between vitamin D levels and leptin and adiponectin levels as obesity markers was conducted using Spearman correlation and linear regression adjusting for the aforementioned covariates; results of the linear regression were expressed as standardized coefficients. Statistical significance was considered for a two-sided test with p < 0.05.

### Ethical statement

The institutional Ethics Committee of the University of Lausanne, which afterwards became the Ethics Commission of Canton Vaud (https://www.cer-vd.ch) approved the baseline CoLaus study (reference 16/03, decisions of 13th January and 10th February 2003). The study was performed in agreement with the Helsinki declaration and its former amendments, and in accordance with the applicable Swiss legislation. All participants gave their signed informed consent before entering the study. Data analysis was conducted in Switzerland and no data was shared with outside groups.

## Results

### Characteristics of participants

Of the initial 6733 participants, 248 were excluded. The detailed reasons for exclusion are provided in supplementary Fig. 1 and the characteristics of excluded and eligible participants are provided in supplementary Table 1. Excluded participants were younger, less frequently born in Switzerland, had a higher educational level, were less frequently former smokers, less physically active and took vitamin D supplements less frequently than included participants.

The characteristics of the 6485 participants by sex are summarized in Table 1. Women were older, had a lower educational level, smoked less, and took vitamin D supplements more frequently. Women also had higher levels of vitamin D and presented less frequently with vitamin D deficiency. Regarding anthropometric markers, women had higher body fat percentage levels but lower levels for all other anthropometric markers than men (Table 1).

**Table 1:** Characteristics of the participants at baseline, by sex, CoLaus|PsyCoLaus study, Lausanne, 2003–2006.

	Women (N = 3401)	Men (N = 3084)	P-value
Age (years)	53.1 ± 10.7	52.3 ± 10.7	0.001
Born in Switzerland (%)	2108 (62.0)	1851 (60.0)	0.106
**Education (%)**			< 0.001
University	540 (15.9)	720 (23.4)	
High school	853 (25.1)	699 (22.7)	
Apprenticeship	1190 (35.0)	1136 (36.8)	
Mandatory	818 (24.1)	529 (17.2)	
**Smoking (%)**			< 0.001
Never	1621 (47.7)	996 (32.3)	
Former	944 (27.8)	1185 (38.4)	
Current	836 (24.6)	903 (29.3)	
Physically active (%)	1865 (54.8)	1578 (51.2)	0.003
**Vitamin D supplement (%)**			
Specific	205 (6.0)	29 (0.9)	< 0.001
Overall	425 (12.5)	182 (5.9)	< 0.001
Vitamin D (nmol/L) median [IQR]	46.2 [30.8–63.4]	43.5 [28.5–60.8]	< 0.001^§^
	48.4 ± 22.6	46.2 ± 22.6	< 0.001
**Vitamin D categories (%)**			0.001
Normal	428 (12.6)	338 (11.0)	
Insufficiency	1078 (31.7)	892 (28.9)	
Deficiency	1895 (55.7)	1854 (60.1)	
**Anthropometry**			
Body mass index (kg/m^2^)	25.1 ± 4.8	26.5 ± 4.0	< 0.001
**Body mass index categories (%)**			< 0.001
Underweight	81 (2.4)	21 (0.7)	
Normal	1869 (55.0)	1143 (37.1)	
Overweight	963 (28.3)	1414 (45.9)	
Obesity	488 (14.4)	506 (16.4)	
Waist (cm)	83.4 ± 12.3	95.5 ± 11.1	< 0.001
Abdominal obesity (%)	1115 (32.8)	798 (25.9)	< 0.001
Hip (cm)	100.6 ± 10.1	102.8 ± 7.9	< 0.001
Waist to hip ratio	0.83 ± 0.07	0.93 ± 0.06	< 0.001
Waist to height ratio	0.51 ± 0.08	0.55 ± 0.07	< 0.001
High waist to height ratio (%)	1686 (49.6)	2312 (75.0)	< 0.001
Body fat percentage (%)	34.3 ± 8.2	23.7 ± 6.0	< 0.001
Conicity index	1.20 ± 0.10	1.29 ± 0.08	< 0.001
High conicity index (%)	1863 (54.8)	2121 (68.8)	< 0.001
Body roundness index	3.7 ± 1.7	4.4 ± 1.4	< 0.001
Body shape index	0.077 ± 0.005	0.081 ± 0.004	< 0.001
**Adipokines**			
Leptin (ng/mL)	14 [8.2–23]	6.4 [3.9–10.7]	< 0.001^§^
Adiponectin (μg/mL)	10.6 [6.9–15.5]	6.2 [4.1–9.2]	< 0.001^§^

Results are expressed as average ± standard deviation or median and interquartile range (IQR) and as number of participants and (column percentage). Between-group comparisons performed using t-test or Kruskal–Wallis test (§) for continuous variables and chi-square for categorical variables.

### Associations between vitamin D levels and anthropometric markers

The levels of vitamin D according to categories of anthropometric markers are summarized in Table 2. On bivariate analysis, and regardless of the anthropometric markers considered, participants with high adiposity levels had lower vitamin D levels than participants with normal adiposity measures. Those findings were further confirmed by multivariate analysis, where a linear trend for a decrease in vitamin D levels with increasing adiposity was found, except for BMI in men (Table 2)*.* Those findings were further confirmed after excluding participants taking vitamin D supplements (supplementary Table 2).

**Table 2:** Bivariate and multivariate comparisons of total vitamin D levels according to adiposity categories, overall and stratified by sex, CoLaus|PsyCoLaus study, Lausanne.

	N	Overall			Women			Men	
		Bivariate	Multivariate	N	Bivariate	Multivariate	N	Bivariate	Multivariate
**Body mass index**									
Underweight	102	56.5 ± 29.9	52.6 ± 1.9	81	59.4 ± 30.1	55.9 ± 2.2	21	45.3 ± 26.8	42.4 ± 3.9
Normal	3012	50.2 ± 23.3	49.5 ± 0.4	1869	51.4 ± 23.2	51.0 ± 0.5	1143	48.1 ± 23.4	47.3 ± 0.5
Overweight	2377	46.3 ± 21.5	46.6 ± 0.4	963	46.1 ± 20.5	46.1 ± 0.6	1414	46.4 ± 22.2	46.8 ± 0.5
Obesity	994	40.6 ± 20.3	42.1 ± 0.6	488	39.6 ± 19.7	41.8 ± 0.9	506	41.4 ± 20.9	42.2 ± 0.8
*p*-value		< 0.001	< 0.001*		< 0.001	< 0.001*		< 0.001	0.945*
**Abdominal obesity**									
Normal	4572	49.1 ± 23.0	49.0 ± 0.3	2286	50.7 ± 23.1	50.5 ± 0.4	2286	47.5 ± 22.9	47.3 ± 0.4
Obesity	1913	43.3 ± 21.1	43.5 ± 0.4	1115	43.8 ± 20.9	44.2 ± 0.6	798	42.5 ± 21.3	43.2 ± 0.7
*p-value*		< 0.001	< 0.001		< 0.001	< 0.001		< 0.001	< 0.001
**Waist to height ratio**									
Normal	2487	51 ± 23.9	50.4 ± 0.4	1715	51.7 ± 23.5	51.3 ± 0.5	772	49.5 ± 24.7	48.5 ± 0.7
Obesity	3998	45.1 ± 21.5	45.5 ± 0.3	1686	45.1 ± 21.1	45.5 ± 0.5	2312	45.1 ± 21.7	45.4 ± 0.4
*p-value*		< 0.001	< 0.001		< 0.001	< 0.001		< 0.001	< 0.001
**Conicity index**									
Normal	2501	49.8 ± 23.2	49.6 ± 0.4	1538	50.2 ± 22.7	50.1 ± 0.5	963	49.3 ± 23.9	48.9 ± 0.6
Elevated	3984	45.8 ± 22.1	46.0 ± 0.3	1863	47.0 ± 22.4	47.0 ± 0.5	2121	44.8 ± 21.8	45.0 ± 0.4
*p-value*		< 0.001	< 0.001		< 0.001	< 0.001		< 0.001	< 0.001
**BRI quartiles**									
First	1625	51.6 ± 24.1	51.0 ± 0.5	854	53.1 ± 23.9	52.7 ± 0.7	772	49.5 ± 24.7	48.8 ± 0.7
Second	1622	49.3 ± 22.8	49.2 ± 0.5	861	50.3 ± 23.1	50.3 ± 0.7	772	47.6 ± 22.0	47.6 ± 0.7
Third	1622	46.6 ± 21.4	46.1 ± 0.5	836	47.7 ± 21.1	47.5 ± 0.7	773	45.4 ± 21.5	45.0 ± 0.7
Fourth	1616	42.1 ± 21.0	43.1 ± 0.5	850	42.5 ± 20.9	43.1 ± 0.7	767	42.4 ± 21.4	43.4 ± 0.7
*p-value*		< 0.001	< 0.001*		< 0.001	< 0.001*		< 0.001	< 0.001*
**ABSI quartiles**									
First	1622	48.8 ± 22.4	48.6 ± 0.5	851	48.7 ± 22.2	49.0 ± 0.7	771	49.2 ± 23.7	49.4 ± 0.7
Second	1621	48.2 ± 22.7	48.2 ± 0.5	850	47.8 ± 21.8	47.7 ± 0.7	771	47.1 ± 21.8	46.6 ± 0.7
Third	1621	47.5 ± 22.8	47.4 ± 0.5	850	48.9 ± 23.5	49.3 ± 0.7	771	45.0 ± 22.3	45.4 ± 0.7
Fourth	1621	45.0 ± 22.4	45.3 ± 0.5	850	48.3 ± 23.0	47.6 ± 0.7	771	43.6 ± 22.1	43.6 ± 0.7
*p-value*		< 0.001	< 0.001*		0.805	0.426*		< 0.001	< 0.001*

*p-value for linear trend. BRI, body roundness index; ABSI, a body shape index. Results are expressed in nmol/L of vitamin D and as mean standard ± deviation for bivariate analyses or as adjusted mean ± standard error for multivariate analyses. Statistical analysis using ANOVA. Multivariate analysis adjusting for age (continuous), nationality (Swiss, other), month, smoking categories (never, former, current), vitamin D supplementation (yes, no) and physical activity (yes, no); for the overall analysis, adjustment on sex (men, women) was also performed.

**Figure 1:**
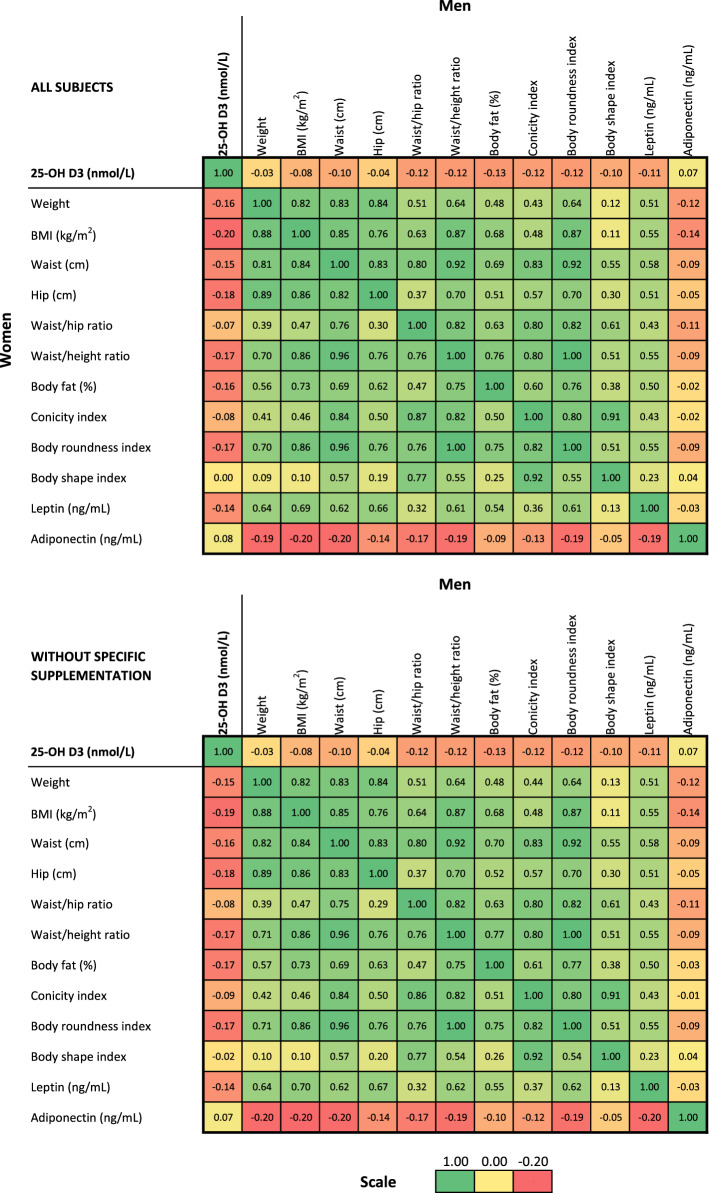
Bivariate non-parametric Spearman correlations between vitamin D levels and anthropometric markers, stratified by gender, CoLaus|PsyCoLaus study, Lausanne. Top panel: all participants. Bottom panel: participants with prescribed supplemental vitamin D excluded. Within each panel, correlations are provided in the lower left for women and in the top right for men.

The bivariate Spearman correlations between vitamin D levels and anthropometric markers are summarized in Fig. 1. Negative correlations were found for all anthropometric markers. With the exception of the correlations between vitamin D and ABSI (in women) and weight (in men), all correlations were statistically significant (p < 0.05). Similar findings were obtained after excluding participants taking vitamin D supplements (Fig. 1).

The results of the stepwise linear regression analysis are summarized in Table 3. After adjusting for major confounders, % body fat was consistently and negatively associated with vitamin D levels overall and for both sexes. Associations were also found for WHtR (overall), BMI (women) and conicity index (men). Similar findings were obtained after excluding participants taking vitamin D supplements (supplementary Table 3) or adjusting for CRP levels (not shown).

**Table 3:** Results of the stepwise linear regression to assess the anthropometric markers most associated with vitamin D levels, overall and stratified by sex, CoLaus|PsyCoLaus study, Lausanne.

	Overall	Women	Men
Weight (kg)	–	–	–
BMI (kg/m^2^)	–	−3.62 (− 4.62; − 2.62)	–
Waist (cm)	–	–	–
Hip (cm)	–	–	–
Waist/hip ratio	–	–	–
Waist/height ratio	−2.78 (−3.51; −2.05)	–	–
Body fat (%)	−2.65 (−3.58; −1.72)	−1.84 (−2.92; −0.75)	−1.79 (−2.68; −0.91)
Conicity index	–	–	−2.63 (−3.46; −1.80)
Body roundness index	–	–	–
Body shape index	–	–	–

Results are expressed as slope and (95% confidence interval) for the markers retained. All anthropometric markers were standardized (i.e., zero average and unit standard deviation) before the stepwise regression. –, not retained.

### Association between vitamin D deficiency levels and anthropometric markers

There were 1895 (55.7%) of women and 1854 (60.1%) men presenting with vitamin D deficiency. Table 4 presents the bivariate and multivariate analysis of the associations between vitamin D deficiency and the different measures of adiposity. The likelihood of vitamin D deficiency increased with increasing adiposity levels; the sole exceptions were no associations with ABSI (women) and BMI (men). Similar findings were obtained after excluding participants taking vitamin D supplements (supplementary Table 4).

**Table 4:** Bivariate and multivariate associations between vitamin D deficiency (i.e. defined as < 30 ng/mL or < 50 nmol/l) and adiposity measures, overall and stratified by sex, CoLaus|PsyCoLaus study, Lausanne.

	N	Overall			Women			Men	
		Bivariate	Multivariate	N	Bivariate	Multivariate	N	Bivariate	Multivariate
**Body mass index**									
Underweight	102	76 (74.5)	1.10 (0.70–1.72)	81	35 (43.2)	1.01 (0.61–1.67)	21	13 (61.9)	1.74 (0.63–4.83)
Normal	3012	2566 (85.2)	1 (ref.)	1869	932 (49.9)	1 (ref.)	1143	645 (56.4)	1 (ref.)
Overweight	2377	2141 (90.1)	1.39 (1.22–1.58)	963	584 (60.6)	1.65 (1.38–1.98)	1414	854 (60.4)	1.10 (0.91–1.33)
Obesity	994	936 (94.2)	1.98 (1.66–2.37)	488	344 (70.5)	2.33 (1.82–2.97)	506	342 (67.6)	1.55 (1.18–2.03)
*p-value*		< 0.001	0.004*		< 0.001	< 0.001*		< 0.001	0.873*
**Abdominal obesity**									
Normal	4572	2508 (54.9)	1 (ref.)	2286	1185 (51.8)	1 (ref.)	2286	1323 (57.9)	1 (ref.)
Obesity	1913	1241 (64.9)	1.65 (1.45–1.88)	1115	710 (63.7)	1.75 (1.48–2.08)	798	531 (66.5)	1.45 (1.18–1.78)
*p-value*		< 0.001	< 0.001		< 0.001	< 0.001		< 0.001	< 0.001
**Waist to height ratio**									
Normal	2487	1283 (51.6)	1 (ref.)	1715	866 (50.5)	1 (ref.)	772	417 (54.0)	1 (ref.)
Obesity	3998	2466 (61.7)	1.53 (1.34–1.73)	1686	1029 (61.0)	1.59 (1.35–1.87)	2312	1437 (62.2)	1.39 (1.13–1.71)
*p-value*		< 0.001	< 0.001		< 0.001	< 0.001		< 0.001	< 0.001
**Conicity index**									
Normal	2501	1331 (53.2)	1 (ref.)	1538	807 (52.5)	1 (ref.)	963	524 (54.4)	1 (ref.)
Elevated	3984	2418 (60.7)	1.43 (1.26–1.62)	1863	1088 (58.4)	1.35 (1.15–1.58)	2121	1330 (62.7)	1.50 (1.23–1.83)
*p-value*		< 0.001	< 0.001		0.001	< 0.001		< 0.001	< 0.001
**BRI quartiles**									
First	1625	818 (50.3)	1 (ref.)	854	399 (46.7)	1 (ref.)	772	417 (54.0)	1 (ref.)
Second	1622	888 (54.8)	1.18 (1.01–1.39)	861	467 (54.2)	1.41 (1.14–1.75)	772	442 (57.3)	1.13 (0.89–1.44)
Third	1622	959 (59.1)	1.53 (1.29–1.82)	836	471 (56.3)	1.60 (1.28–1.99)	773	481 (62.2)	1.56 (1.21–2.00)
Fourth	1616	1084 (67.1)	2.06 (1.72–2.47)	850	558 (65.7)	2.41 (1.90–3.06)	767	514 (67.0)	1.73 (1.32–2.26)
*p-value*		< 0.001	< 0.001*		< 0.001	< 0.001*		< 0.001	< 0.001*
**ABSI quartiles**									
First	1622	904 (55.7)	1 (ref.)	851	472 (55.5)	1 (ref.)	771	422 (54.7)	1 (ref.)
Second	1621	907 (56.0)	0.99 (0.84–1.16)	850	485 (57.1)	1.14 (0.92–1.41)	771	442 (57.3)	1.27 (1.00–1.62)
Third	1621	937 (57.8)	1.09 (0.92–1.30)	850	469 (55.2)	1.00 (0.80–1.23)	771	490 (63.6)	1.66 (1.29–2.13)
Fourth	1621	1001 (61.8)	1.29 (1.07–1.55)	850	469 (55.2)	1.09 (0.88–1.37)	771	500 (64.9)	1.87 (1.43–2.44)
*p-value*		0.001	0.005*		0.839	0.697*		< 0.001	< 0.001*

*p-value for linear trend for multivariate analysis. BRI, body roundness index; ABSI, a body shape index. Results are expressed as number of participants and row (%) of vitamin D deficiency for bivariate analyses or as adjusted odds ratio (95% confidence interval) for vitamin D insufficiency. Bivariate analysis using chi-square. Multivariate analysis using logistic regression adjusting for age (continuous), nationality (Swiss, other), month, smoking categories (never, former, current), vitamin D supplementation (yes, no) and physical activity (yes, no); for the overall analysis, adjustment on sex (men, women) was also performed.

### Association between vitamin D levels and adipokines

The associations between vitamin D, leptin and adiponectin levels are summarized in Fig. 1 (bivariate) and supplementary Table 5 (multivariate). In both univariate and multivariate analyses, leptin levels were negatively associated with vitamin D levels. Adipokine levels were positively associated with vitamin D levels in women, while no association was found in men. Similar findings were obtained after excluding participants taking vitamin D supplements (Fig. 1 and supplementary Table 5).

## Discussion

To our knowledge, this is one of the most comprehensive study in a large population-based cohort of apparently healthy subjects associating vitamin D levels with a wide array of obesity markers. Our results show a negative association between various adiposity measurements and vitamin D levels, and that total body fatness is the adiposity marker most associated with vitamin D levels. Our results add further evidence to the hypothesis that low vitamin D levels might be explained by the sequestration of the vitamin by excess body fat stores.

### Associations between vitamin D levels and anthropometric markers

Negative associations between vitamin D levels and all markers of adiposity were found, the sole exception being BMI in men, where underweight participants had vitamin D levels close to those of participants with obesity. A possible explanation is that underweight men might have reduced vitamin D dietary intake and/or be affected by pathophysiological conditions affecting vitamin D metabolism, but this hypothesis remains to be assessed. Still, our findings are consistent with the previous literature. Inverse associations have been reported between vitamin D levels and abdominal and visceral fat^26^; waist circumference and BRI^27^; the visceral adiposity index^28^, and with both total and regional adiposity^29^. Also, De Pergola et al., found that vitamin D circulating levels were progressively lower with the increase of fat mass in a cohort of healthy overweight and subjects with obesity^5^.

WC, WHR and WHtR are traditional indicators of abdominal obesity. BRI and ABSI have recently been proposed as novel anthropometric measurements and are positively correlated with visceral adiposity^10,21,27^. Conversely, CI is rarely used as a measure to assess adiposity, despite being a good predictor of abdominal adiposity^8^. Notwithstanding, total body fat was the sole anthropometric marker consistently associated with low vitamin D levels, suggesting that it is the total amount of body fat, rather than localized or unevenly distributed body fat, that are associated with vitamin D levels. Our results thus add further evidence to the hypothesis that low levels of vitamin D in individuals with obesity can be explained by the sequestration of vitamin D by adipose tissue or by simple volumetric dilution in adipose tissue^30^.

### Association between vitamin D deficiency levels and anthropometric markers

Over half of the sample presented with vitamin D deficiency, hypovitaminosis D (i.e. insufficiency or deficiency) being more frequent in men than in women. Our findings do not replicate recent studies showing that hypovitaminosis D is more prevalent in women than in men^10,31,32^. Possible explanations include the fact that the women in this study were less frequently were less frequently diagnosed with obesity than men, or an unhealthier dietary intake in men.

Overall, our results show that individuals with higher levels of adiposity are more predisposed to reduced serum concentrations of vitamin D. This reduced bioavailability would trigger a hypothalamic action, stimulating food intake and reducing energy expenditure^33^. Chronic inflammation linked to obesity could also explain hypovitaminosis D^34^ due to adipose tissue macrophage infiltration and production of proinflammatory adipokines^35^. It has also been postulated that the increase in adiposity would be promoted by resistance to stimulation of lipolysis by catecholamines and natriuretic peptide in people with obesity^36^. Furthermore, this imbalance may lead to reduced release of vitamin D from fat depots, given the fat-soluble nature of vitamin D^36^.

Importantly, it is unclear whether increasing vitamin D supplementation to correct low vitamin D levels in subjects with increased adiposity is the best option. One study demonstrated that adapting supplementation to both vitamin D deficit and adiposity levels would be more efficient^37^, but further studies are needed to confirm this finding. Besides, improvements in vitamin D levels have been associated with more weight loss, a result which can be partly attributed to the anti-inflammatory effects of vitamin D^33^.

### Association between vitamin D levels and adipokines

Our study showed that vitamin D levels were inversely associated with leptin and positively associated with adiponectin. Our findings are in agreement with those of Gangloff et al.^38^, who reported a negative correlation vitamin D levels and leptin. The authors also reported that a decrease in visceral adipose tissue volume and corresponding decrease in leptin levels was associated with an increase in plasma vitamin D concentrations.

Studies have shown that vitamin D affects energy homeostasis by directly regulating leptin expression. However, the exact in vivo effect of vitamin D on leptin expression in humans is controversial^11^. Some studies have suggested that vitamin D can improve adipose tissue inflammation and suppress expression of leptin^39^. A recent study demonstrated that vitamin D acts through its receptor (VDR) to inhibit inflammatory pathways and the expression of adipokines in human adipocytes^12^. Overall, these findings suggest that improving vitamin D status in people with obesity may decrease adipose inflammation, which may contribute to reduce risks of obesity-associated pathophysiological processes. Although more recent studies point to CRP as a marker of inflammation associated with vitamin D levels, our results remained unchanged after adjusting for CRP.

### Strengths and limitations

This study was carried out in a representative sample of the population and included a large panel of adiposity markers.

Some limitations should be acknowledged. First, the analysis was conducted in an urban Swiss population and the participants were mostly Caucasian. The results might not be generalizable to other countries. Still, our results are concordant with ones reported in other populations. Second, fat mass was measured by bioimpedance with the known limitations of this technique to accurately quantify adipose tissue particularly using a single time point measurement. Third, our cross-sectional study does not allow to draw any potential causality relationships between the investigated variables (i.e. whether it is obesity that causes vitamin D deficiency or vice versa). However, a prospective study is envisaged. Fourthly, we did not use different cutoff points for vitamin D according to seasonality as indicated in other studies^40^. Still, we adjusted the analysis considering the month. We chose this methodology as changing the threshold according to season would be difficult to apply in clinical practice.

## Conclusions

Our data show a negative association between vitamin D levels and most adiposity markers and that people with excess adiposity are more likely exhibit to present with vitamin D deficiency as measured by total circulating 25-hydroxyvitamin D. Total fat mass is the adiposity marker most consistently associated with decreased vitamin D levels. Monitoring the levels of vitamin D in people with overweight/obesity is essential.

## Supplementary Information

Supplementary Tables.

Supplementary Figure 1.

## Data Availability

The CoLaus|PsyCoLaus cohort data used in this study cannot be fully shared as they contain potentially sensitive patient information. As discussed with the competent authority, the Research Ethic Committee of the Canton of Vaud, transferring or directly sharing this data would be a violation of the Swiss legislation aiming to protect the personal rights of participants. Non-identifiable, individual-level data are available for interested researchers, who meet the criteria for access to confidential data sharing, from the CoLaus Datacenter (CHUV, Lausanne, Switzerland). Instructions for gaining access to the CoLaus data used in this study are available at https://www.colaus-psycolaus.ch/professionals/how-to-collaborate/.
