# Supplementary material for: Association between anthropometric markers of adiposity, adipokines and vitamin D levels
Source: Sci Rep. 2022 Sep 14;12:15435. doi: 10.1038/s41598-022-19409-9 (PMC9474508; doi:10.1038/s41598-022-19409-9)
Supplement: Supplementary file 1 — Supplementary Tables. [file 41598_2022_19409_MOESM1_ESM.docx]

**Supplementary information**

**Supplementary table 1**: comparison between excluded and included participants, CoLaus|PsyCoLaus study, Lausanne.

|  | **Included (N=6485)** | **Excluded (N=248)** | **P-value** |
| --- | --- | --- | --- |
| Age (years) | 52.7 ± 10.7 | 50.4 ± 10.8 | <0.001 |
| Women (%) | 3401 (52.4) | 143 (57.7) | 0.106 |
| Born in Switzerland (%) | 3959 (61.1) | 72 (29.3) | <0.001 |
| Education (%) |  |  | <0.001 |
| University | 1260 (19.4) | 60 (25.6) |  |
| High school | 1552 (23.9) | 73 (31.2) |  |
| Apprenticeship | 2326 (35.9) | 51 (21.8) |  |
| Mandatory | 1347 (20.8) | 50 (21.4) |  |
| Smoking (%) |  |  | 0.003 |
| Never | 2617 (40.4) | 115 (47.5) |  |
| Former | 2129 (32.8) | 54 (22.3) |  |
| Current | 1739 (26.8) | 73 (30.2) |  |
| Physically active (%) | 3443 (53.1) | 64 (41.6) | 0.005 |
| Vitamin D supplement (%) |  |  |  |
| Specific | 234 (3.6) | 5 (2.0) | 0.184 |
| Overall | 607 (9.4) | 11 (4.4) | 0.008 |

Results are expressed as average ± standard deviation or as number of participants and (percentage). Between-group comparisons performed using t-test for continuous variables and chi-square for categorical variables.

**Supplementary table 2**: bivariate and multivariate comparisons of vitamin D levels according to obesity and conicity categories, overall and stratified by gender, CoLaus|PsyCoLaus study, Lausanne. Participants with prescribed supplemental vitamin D excluded.

|  |  | **Overall** |  |  | **Women** |  |  | **Men** |  |
| --- | --- | --- | --- | --- | --- | --- | --- | --- | --- |
|  | **N** | **Bivariate** | **Multivariate** | **N** | **Bivariate** | **Multivariate** | **N** | **Bivariate** | **Multivariate** |
| Body mass index |  |  |  |  |  |  |  |  |  |
| Underweight | 92 | 54.4 ± 29.2 | 51.0 ± 2.0 | 72 | 57.0 ± 29.3 | 53.6 ± 2.3 | 20 | 45.2 ± 27.5 | 42.6 ± 4.0 |
| Normal | 2884 | 49.3 ± 23.0 | 48.8 ± 0.4 | 1750 | 50.1 ± 22.7 | 49.8 ± 0.5 | 1134 | 47.9 ± 23.3 | 47.1 ± 0.5 |
| Overweight | 2307 | 45.8 ± 21.4 | 46.0 ± 0.4 | 906 | 45.0 ± 20.0 | 44.9 ± 0.6 | 1401 | 46.3 ± 22.2 | 46.7 ± 0.5 |
| Obesity | 968 | 40.2 ± 20.3 | 41.6 ± 0.6 | 468 | 38.8 ± 19.5 | 40.8 ± 0.9 | 500 | 41.5 ± 20.9 | 42.3 ± 0.8 |
| *p-value ** |  | <0.001 | <0.001 * |  | <0.001 | <0.001 * |  | <0.001 | 0.906 * |
| Abdominal obesity |  |  |  |  |  |  |  |  |  |
| Normal | 4411 | 48.4 ± 22.7 | 48.2 ± 0.3 | 2148 | 49.4 ± 22.5 | 49.2 ± 0.4 | 2263 | 47.4 ± 22.8 | 47.1 ± 0.4 |
| Obesity | 1840 | 42.5 ± 20.9 | 42.9 ± 0.5 | 1048 | 42.6 ± 20.5 | 43.0 ± 0.6 | 792 | 42.4 ± 21.3 | 43.2 ± 0.7 |
| *p-value* |  | <0.001 | <0.001 |  | <0.001 | <0.001 |  | <0.001 | <0.001 |
| Waist to height ratio |  |  |  |  |  |  |  |  |  |
| Normal | 2381 | 50.1 ± 23.5 | 49.5 ± 0.4 | 1613 | 50.5 ± 23.0 | 50.0 ± 0.5 | 768 | 49.3 ± 24.6 | 48.3 ± 0.7 |
| Obesity | 3870 | 44.5 ± 21.3 | 44.9 ± 0.3 | 1583 | 43.8 ± 20.6 | 44.3 ± 0.5 | 2287 | 45.0 ± 21.7 | 45.4 ± 0.4 |
| *p-value* |  | <0.001 | <0.001 |  | <0.001 | <0.001 |  | <0.001 | <0.001 |
| Conicity index |  |  |  |  |  |  |  |  |  |
| Normal | 2419 | 49.2 ± 22.9 | 48.9 ± 0.4 | 1459 | 49.2 ± 22.3 | 48.9 ± 0.5 | 960 | 49.3 ± 23.8 | 48.8 ± 0.6 |
| Elevated | 3832 | 45.1 ± 21.8 | 45.3 ± 0.3 | 1737 | 45.5 ± 21.8 | 45.7 ± 0.5 | 2095 | 44.7 ± 21.8 | 44.9 ± 0.4 |
| *p-value* |  | <0.001 | <0.001 |  | <0.001 | <0.001 |  | <0.001 | <0.001 |
| BRI quartiles |  |  |  |  |  |  |  |  |  |
| First | 1549 | 50.6 ± 23.7 | 50.1 ± 0.5 | 800 | 51.8 ± 23.4 | 51.4 ± 0.7 | 768 | 49.3 ± 24.6 | 48.5 ± 0.7 |
| Second | 1570 | 48.6 ± 22.5 | 48.4 ± 0.5 | 813 | 49.1 ± 22.6 | 48.9 ± 0.7 | 765 | 47.6 ± 22.0 | 47.6 ± 0.7 |
| Third | 1560 | 46.0 ± 21.4 | 45.6 ± 0.5 | 781 | 46.5 ± 20.5 | 46.4 ± 0.7 | 762 | 45.3 ± 21.5 | 45.0 ± 0.7 |
| Fourth | 1572 | 41.5 ± 20.7 | 42.4 ± 0.5 | 802 | 41.2 ± 20.4 | 41.9 ± 0.7 | 760 | 42.2 ± 21.4 | 43.4 ± 0.7 |
| *p-value* |  | <0.001 | <0.001 * |  | <0.001 | <0.001 * |  | <0.001 | <0.001 * |
| BSI quartiles |  |  |  |  |  |  |  |  |  |
| First | 1549 | 47.8 ± 22.0 | 47.8 ± 0.5 | 805 | 47.6 ± 21.7 | 47.8 ± 0.7 | 768 | 49.1 ± 23.6 | 49.3 ± 0.7 |
| Second | 1569 | 47.7 ± 22.6 | 47.7 ± 0.5 | 805 | 46.7 ± 21.4 | 46.4 ± 0.7 | 762 | 47.1 ± 21.9 | 46.6 ± 0.7 |
| Third | 1568 | 47.0 ± 22.7 | 46.7 ± 0.5 | 803 | 47.8 ± 23.2 | 48.2 ± 0.7 | 762 | 44.7 ± 22.2 | 45.1 ± 0.7 |
| Fourth | 1565 | 44.1 ± 21.9 | 44.4 ± 0.5 | 783 | 46.5 ± 22.2 | 46.3 ± 0.7 | 763 | 43.5 ± 22.1 | 43.5 ± 0.7 |
| *p-value* |  | <0.001 | <0.001 * |  | 0.588 | 0.116 |  | <0.001 | <0.001 * |

*, p-value for linear trend. BRI, body roundness index; BSI, body shape index. Results are expressed in nmol/L of vitamin D and as mean standard±deviation for bivariate analyses or as adjusted mean±standard error for multivariate analyses. Statistical analysis using ANOVA. Multivariate analysis adjusting for age (continuous), nationality (Swiss, other), month, smoking categories (never, former, current) and physical activity (yes, no); for the overall analysis, adjustment on gender (men, women) was also performed.

**Supplementary table 3**: results of the stepwise linear regression to assess the anthropometric markers most associated with vitamin D levels, overall and stratified by gender, CoLaus|PsyCoLaus study, Lausanne. Participants with prescribed supplemental vitamin D excluded.

|  | **Overall** | **Women** | **Men** |
| --- | --- | --- | --- |
| Weight (kg) | - | - | - |
| BMI (kg/m^2^) | - | -3.29 (-4.29; -2.28) | - |
| Waist (cm) | - | - | - |
| Hip (cm) | - | - | 1.27 (0.40; 2.14) |
| Waist/hip ratio | - | - | - |
| Waist/height ratio | -2.65 (-3.47; -1.84) | - | - |
| Body fat (%) | -2.78 (-3.82; -1.74) | -1.65 (-2.75; -0.56) | -2.39 (-3.34; -1.43) |
| Conicity index | - | - | -3.07 (-3.97; -2.16) |
| Body roundness index | - | - | - |
| Body shape index | - | - | - |

Results are expressed as slope and (95% confidence interval) for the markers retained. All anthropometric markers were standardized (i.e., zero average and unit standard deviation) before the stepwise regression. -, not retained.

**Supplementary table 4**: bivariate and multivariate comparisons of vitamin D deficiency according to obesity and conicity categories, overall and stratified by gender, CoLaus|PsyCoLaus study, Lausanne. Participants with prescribed supplemental vitamin D excluded.

|  | **N** | **Bivariate** | **Multivariate** | **N** | **Bivariate** | **Multivariate** | **N** | **Bivariate** | **Multivariate** |
| --- | --- | --- | --- | --- | --- | --- | --- | --- | --- |
| Body mass index |  |  |  |  |  |  |  |  |  |
| Underweight | 92 | 45 (48.9) | 1.05 (0.66 - 1.69) | 72 | 33 (45.8) | 1.00 (0.59 - 1.7) | 20 | 12 (60.0) | 1.47 (0.52 - 4.18) |
| Normal | 2884 | 1553 (53.9) | 1 (ref.) | 1750 | 910 (52.0) | 1 (ref.) | 1134 | 643 (56.7) | 1 (ref.) |
| Overweight | 2307 | 1420 (61.6) | 1.39 (1.22 - 1.58) | 906 | 570 (62.9) | 1.66 (1.38 - 2.00) | 1401 | 850 (60.7) | 1.09 (0.90 - 1.32) |
| Obesity | 968 | 677 (69.9) | 1.97 (1.64 - 2.36) | 468 | 339 (72.4) | 2.35 (1.82 - 3.02) | 500 | 338 (67.6) | 1.53 (1.17 - 2.00) |
| *p-value ** |  | <0.001 | 0.004 |  | <0.001 | <0.001 * |  | 0.001 | 0.902 * |
| Abdominal obesity |  |  |  |  |  |  |  |  |  |
| Normal | 4411 | 2473 (56.1) | 1 (ref.) | 2148 | 1158 (53.9) | 1 (ref.) | 2263 | 1315 (58.1) | 1 (ref.) |
| Obesity | 1840 | 1222 (66.4) | 1.66 (1.45 - 1.89) | 1048 | 694 (66.2) | 1.77 (1.48 - 2.11) | 792 | 528 (66.7) | 1.44 (1.17 - 1.78) |
| *p-value* |  | <0.001 | <0.001 |  | <0.001 | <0.001 |  | <0.001 | <0.001 |
| Waist to height ratio |  |  |  |  |  |  |  |  |  |
| Normal | 2381 | 1263 (53.0) | 1 (ref.) | 1613 | 847 (52.5) | 1 (ref.) | 768 | 416 (54.2) | 1 (ref.) |
| Obesity | 3870 | 2432 (62.8) | 1.52 (1.34 - 1.73) | 1583 | 1005 (63.5) | 1.59 (1.34 - 1.87) | 2287 | 1427 (62.4) | 1.39 (1.13 - 1.71) |
| *p-value* |  | <0.001 | <0.001 |  | <0.001 | <0.001 |  | <0.001 | 0.002 |
| Conicity index |  |  |  |  |  |  |  |  |  |
| Normal | 2419 | 1314 (54.3) | 1 (ref.) | 1459 | 792 (54.3) | 1 (ref.) | 960 | 522 (54.4) | 1 (ref.) |
| Elevated | 3832 | 2381 (62.1) | 1.44 (1.27 - 1.63) | 1737 | 1060 (61.0) | 1.36 (1.15 - 1.60) | 2095 | 1321 (63.1) | 1.52 (1.25 - 1.85) |
| *p-value* |  | <0.001 | <0.001 |  | <0.001 | <0.001 |  | <0.001 | <0.001 |
| BRI quartiles |  |  |  |  |  |  |  |  |  |
| First | 1549 | 804 (51.9) | 1 (ref.) | 800 | 390 (48.8) | 1 (ref.) | 768 | 416 (54.2) | 1 (ref.) |
| Second | 1570 | 877 (55.9) | 1.19 (1.01 - 1.40) | 813 | 457 (56.2) | 1.42 (1.14 - 1.77) | 765 | 438 (57.3) | 1.12 (0.88 - 1.43) |
| Third | 1560 | 942 (60.4) | 1.53 (1.28 - 1.81) | 781 | 458 (58.6) | 1.58 (1.26 - 1.98) | 762 | 477 (62.6) | 1.56 (1.21 - 2.01) |
| Fourth | 1572 | 1072 (68.2) | 2.06 (1.71 - 2.48) | 802 | 547 (68.2) | 2.45 (1.91 - 3.13) | 760 | 512 (67.4) | 1.73 (1.32 - 2.27) |
| *p-value* |  | <0.001 | <0.001 * |  | <0.001 | <0.001 * |  | <0.001 | <0.001 * |
| BSI quartiles |  |  |  |  |  |  |  |  |  |
| First | 1549 | 889 (57.4) | 1 (ref.) | 805 | 464 (57.6) | 1 (ref.) | 768 | 420 (54.7) | 1 (ref.) |
| Second | 1569 | 893 (56.9) | 0.98 (0.83 - 1.15) | 805 | 475 (59.0) | 1.13 (0.90 - 1.40) | 762 | 437 (57.4) | 1.27 (0.99 - 1.61) |
| Third | 1568 | 923 (58.9) | 1.10 (0.92 - 1.31) | 803 | 459 (57.2) | 0.98 (0.79 - 1.22) | 762 | 489 (64.2) | 1.69 (1.32 - 2.18) |
| Fourth | 1565 | 990 (63.3) | 1.31 (1.08 - 1.58) | 783 | 454 (58.0) | 1.07 (0.85 - 1.35) | 763 | 497 (65.1) | 1.88 (1.44 - 2.46) |
| *p-value* |  | <0.001 | 0.003 * |  | 0.895 | 0.830 |  | <0.001 | <0.001 * |

* p-value for linear trend for multivariate analysis. BRI, body roundness index; BSI, body shape index. Results are expressed as number of participants (%) of vitamin D deficiency for bivariate analyses or as adjusted odds ratio (95% confidence interval) for vitamin D deficiency. Bivariate analysis using chi-square. Multivariate analysis using logistic regression adjusting for age (continuous), nationality (Swiss, other), month, smoking categories (never, former, current) and physical activity (yes, no); for the overall analysis, adjustment on gender (men, women) was also performed.

**Supplementary table 5**: multivariate associations between vitamin D and leptin or adiponectin levels, overall and stratified by gender, CoLaus|PsyCoLaus study, Lausanne.

|  | **Overall** | **Women** | **Men** |
| --- | --- | --- | --- |
| **All participants** |  |  |  |
| Leptin (ng/mL) | -0.130 | -0.136 | -0.089 |
| Adiponectin (ng/mL) | 0.051 | 0.067 | 0.011 |
| **Excluding vitamin D supplements** |  |  |  |
| Leptin (ng/mL) | -0.121 | -0.125 | -0.087 |
| Adiponectin (ng/mL) | 0.044 | 0.063 | 0.007 |

Results are expressed as standardized regression coefficient. Non-significant (p<0.05) associations are indicated in grey background.
