# Supplementary figures and images for: Association between anthropometric markers of adiposity, adipokines and vitamin D levels
Source: Sci Rep. 2022 Sep 14;12:15435. doi: 10.1038/s41598-022-19409-9 (PMC9474508; doi:10.1038/s41598-022-19409-9)

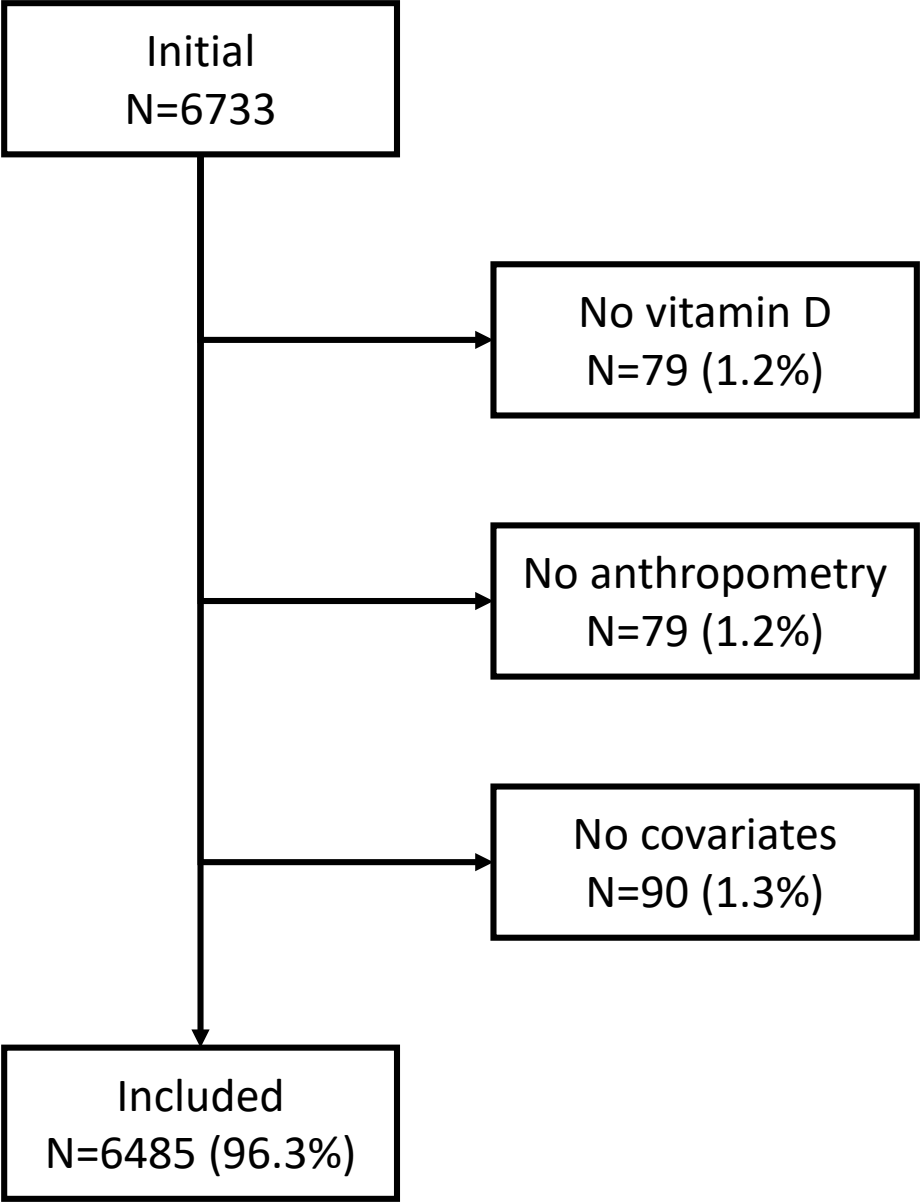

Supplement: Supplementary file 2 — Supplementary Figure 1. [file 41598_2022_19409_MOESM2_ESM.pdf]
